# Development of a patient‐centred conceptual framework of health‐related quality of life in neuromyelitis optica: a qualitative study

**DOI:** 10.1111/hex.12432

**Published:** 2015-11-24

**Authors:** Abigail M. Methley, Kerry Mutch, Perry Moore, Anu Jacob

**Affiliations:** ^1^ School of Psychological SciencesUniversity of ManchesterManchesterLancashireUK; ^2^ Walton Centre NHS Foundation TrustLiverpoolMerseysideUK; ^3^ NMO Clinical ServiceWalton Centre NHS Foundation TrustLiverpoolMerseysideUK; ^4^ Neuropsychology DepartmentWalton Centre NHS Foundation TrustLiverpoolMerseysideUK; ^5^ Cardiff UniversityGlamorganUK

**Keywords:** neurology, neuromyelitis optica, patient‐centred, patient‐reported outcome measure, qualitative, quality of life

## Abstract

**Background:**

Neuromyelitis optica (NMO) is an auto‐immune disease that can cause severe visual and mobility impairments. Research on health‐related quality of life (HRQoL) in NMO is scarce, limiting knowledge on factors influencing HRQoL and support needs.

**Aim:**

This study provides the first qualitative exploration of HRQoL in NMO, conducted to provide a conceptual framework for the development of an NMO patient‐reported outcome measure.

**Method:**

Fifteen people with NMO (aged 18–74; 11 women, 4 men) participated in semi‐structured interviews; data were analysed using constant comparative analysis.

**Results:**

HRQoL in NMO is a multifaceted concept incorporating highly subjective perceptions of normality and meaning. Four major themes were identified: impact of physical symptoms on daily living, utilizing support to achieve independence, expectations for life and meaningful roles in life and purpose.

**Discussion:**

Themes highlighted the importance of perceived normality, and its relationship to attaining life goals comparable to peers, as underpinning evaluations of HRQoL. Many people with severe disability reported a high HRQoL, suggesting the inappropriateness of assuming a negative HRQoL on the basis of an individual's neurological impairment.

**Conclusions:**

These findings further the conceptual understanding of HRQoL in NMO, informing patient‐care approaches and the development of an NMO‐specific patient‐reported outcome measure.

## Introduction

Neuromyelitis Optica (NMO) is an auto‐immune condition affecting the central nervous system. Symptoms begin at any age, with the most common age of onset in the fourth decade^1^; women are predominantly affected (gender ratio of 5 : 1).^2^ The limited epidemiological data suggests an estimated half a million cases worldwide, with 4000 cases in the United States^3^ and a prevalence rate of 7.2–19.6 per million in the United Kingdom,^4^, ^5^ affecting approximately 700 people. In comparison to the approximately 345 000 people living with epilepsy in the UK,^6^ NMO is a very rare neurological condition, with accompanying difficulties regarding lack of public and professional awareness and knowledge, and potentially negative experiences of health‐care services.^7^, ^8^

NMO is characterized by periodic exacerbations of symptoms (relapses) and significant disability that often occurs early in the disease process, compared to a condition with similar clinical features, such as multiple sclerosis. In comparison to other relapsing conditions such as multiple sclerosis^9^ or rheumatoid arthritis^10^ NMO relapses typically have a rapid onset and cause more severe residual neurological disability. Relapses cause transverse myelitis, optic neuritis and brain stem involvement which can cause a wide variety of symptoms.^4^

Treatments, mainly of an immunosuppressive nature, are only partially effective. The aim of treatment is to prevent further relapses as disability accrues with each relapse; however, medications such as immunosuppressants and corticosteroids often cause intolerable and often life‐threatening side‐effects.^11^ The pathogenic mechanisms underlying NMO are only partially understood, although anti‐aquaporin‐4 IgG (an antibody) is present in the sera of up to 70% of patients with NMO and is thought to mediate the disease.^12^

The severity of symptoms, combined with the varied and unpredictable nature of the condition, has a significant impact on individuals' daily lives that can contribute to psychological difficulties in adjusting to life with this condition.^7^ However, as with other neurological conditions, research into lived experience and quality of life (QoL) in NMO has come secondary to investigations of health status, limiting the ability of health‐care professionals to prioritize and influence well‐being in daily living and to evaluate treatment effectiveness from a patient perspective.^13^ As the need to engage people with neurological conditions in complex and subjective medical and health‐care decisions increases with the development of new treatments,^14^ now is a crucial time to expand knowledge on how they define their QoL.

## NMO and health‐related quality of life

In 2006, the World Health Organization concluded that the QoL of people with neurological conditions needed to be significantly improved internationally^15^ and QoL is now appreciated when investigating the value of health‐care services to patients.^16^ QoL has therefore become a major focus of United Kingdom health‐care policy for people with long‐term conditions.^16^

Health‐related quality of life (HRQoL) is a difficult concept to define, being both multidisciplinary and multidimensional;^17^, ^18^ it commonly includes physical, psychological, environmental and social issues, alongside one's level of independence and personal beliefs.^19^ There is no gold standard to define HRQoL.^20^ This multifaceted characteristic has been highlighted in previous research in neurological conditions such as multiple sclerosis, demonstrating physical disability alone does not fully explain satisfaction with life.^21^

A limitation of standardized questionnaire measures of HRQoL is that they constrain participants' ability to identify the factors they perceive to be the major influences on their QoL, or to discuss why.^22^, ^23^ No disease‐specific outcome measure exists for measuring HRQoL in NMO, although NMO has several unique disease related considerations including its rarity, the limitations of medical understanding of this condition and a unique and highly variable clinical course. As part of an on‐going study to develop a patient‐reported outcome measure for NMO, a systematic review of major databases (EMBASE, MEDLINE and PsycInfo) was completed. This did not identify any previous qualitative studies of quality of life in NMO. Considered alongside the limited prior quantitative data on HRQoL in NMO^24^, ^25^, ^26^ this suggests a more exploratory approach to investigating HRQoL for people with NMO is necessary to understand the concepts most salient to HRQoL in affected individuals, rather than assuming validity of existing QoL instruments.

Our aim was therefore to investigate HRQoL in NMO to inform development of a HRQoL conceptual framework and to identify factors relevant to the development of a patient‐reported outcome measure.

## Method

### Patients

Participants were originally recruited as part of a larger study into the lived experience of NMO.^7^ Twenty‐eight participants were invited to participate. Fifteen participants were willing to participate and recruited between 2012 and 2013. Participants completed one interview, at a location of their choosing (their home or hospital outpatient clinic room). A maximum variation purposeful sampling technique was used to aim for a sample of men and women of a variety of ages, ethnicities and levels of disability.^27^ Once analysis had begun, researchers aimed to identify the ‘extreme variations’ (p. 65)^28^ in all concepts. This was achieved by recruiting participants who represented deviant or disconfirming cases (defined as ‘elements in the data that contradict the emerging explanation of the phenomena under study’ (p. 51),^29^ to test and refine devised subthemes and themes by illuminating their boundaries and limitations. Recruitment ceased after the fifteenth interview as researchers felt that all the major concepts had been identified and united into the emerging conceptual framework, that is theoretical saturation had occurred.^28^

All participants had clinically definite NMO (fulfilling Wingerchuck criteria, 2006^30^) and nine demonstrated the specific antibody present in 70% of people with NMO (anti‐aquaporin‐4 IgG positive). For ethical reasons no participants were invited to participate while relapsing (experiencing acute sight loss or mobility impairment). The sample was recruited through a specialist outpatient service and was therefore receiving clinical review on at least an annual basis. Sociodemographic information was collected through a basic demographic questionnaire. Disability information was collected using the Expanded Disability Status Scale^31^; a measure of functioning commonly used in studies investigating neurological conditions e.g. Multiple Sclerosis or NMO. Demographic and clinical characteristics are presented in Table 1. Ethics approval was obtained from the National Research Ethics Committee. All participants gave written informed consent before participating.

**Table 1:** Demographic and clinical characteristics of sample

Age	18–74 years (median age 48 years)
Gender	F : 11; M : 4
Independent living	14 (1 with full time carer)
Ethnicity	White British (*n* = 12), Caribbean (*n* = 1) Chinese (*n* = 1) and Asian (*n* = 1)
Time since onset of NMO symptoms	1.5–34 years (mean 8.7 years, *SD* 17.05 years)
The median number of relapses at time of recruitment	3
Visual impairment	11
Sensory impairment	4
Motor impairment	13. Wheelchair dependent (*n* = 3), used one cane (*n* = 5), partially sighted or blind (*n* = 4)
Median Expanded Disability Status Score (EDSS^17^)	5
Employment status	Retired (*n* = 2), student (*n* = 1), employed (*n* = 3), searching for employment (*n* = 2), finished work due to ill‐health (*n* = 7)

### Measures

Semi‐structured interviews were used to introduce the central topic while providing the flexibility for participants to bring up issues important to them. Participants were asked about what quality of life meant to them, what impact NMO had on their quality of life, and what improved or decreased their quality of life.

Because of the lack of prior literature available and concerns about applying pre‐existing assumptions, the topic guide was kept deliberately broad to ensure that no assumptions were made about HRQoL in the patient sample. Objective measurements regarding the nature of ‘high’/‘low’ or ‘good’/‘bad’ HRQoL were not taken, allowing participants to define their interpretation of HRQoL. All interviews were audio‐recorded and transcribed verbatim before being anonymized. Interviews lasted approximately 30–60 min. All interviews were conducted face‐to‐face by a Specialist Nurse or Psychologist, who were both experienced in qualitative interviewing methods.

### Analysis

Due to limited prior research it was not possible to generate an *a priori* coding framework outlining relevant factors of HRQoL for people with NMO. It was therefore important to use an inductive method based on the principles of grounded theory,^32^ to investigate the themes that arose from the data without imposing a framework onto the data and forcing it to fit pre‐defined categories.

The constant comparison approach (a central aspect of grounded theory) creates codes which are then compared both within and across participants.^33^ This conceptualizes codes into a higher, more encompassing and therefore potentially more theoretical category,^34^ allowing the identification of categories and themes across an entire dataset. The overall process of constant comparison analysis followed the systematic process outlined in Boeije (2002; example theme provided in Supplementary Figure 1).^35^

### Coding

A code was defined as a word or phrase characterizing the crux of a segment of data relevant to the research question.^36^ Codes were derived from the data (*a posteri*) not imposed upon it *a priori*. The coding process began to analyse and order the data,^37^ enabling discussion of the meanings and assumptions attributed to data.

Transcripts were read multiple times while listening to an audio recording of the interview to ensure familiarity with the data. During this process initial codes were made through primarily descriptive open coding (including *in vivo* quotes). Open coding has been defined by Strauss and Corbin (1990, p.61)^37^ as ‘the process of breaking down, examining, comparing, conceptualizing and categorizing data’, providing a descriptive overview of broadly similar sections of the data.

### Categorizing

Selective (axial) coding then aimed to specify a phenomenon or category by defining the environment in which it occurred and surrounding context.^37^ In doing so, coding became more focussed on the key research question,^38^ suggesting potential further sampling needs. Data collection and analysis were contemporaneous.^34^ The constant comparison of codes allowed the identification of recurrent categories and themes which addressed the research question. Where codes described a similar phenomenon a higher more abstract concept called a category was devised.^37^ Category names were relevant to the data and concise and coherent enough to communicate the essence of the data to the reader; some existed in the literature as the concepts associated with established terms communicated the content (and positioning) of the data best. However, care was taken within the research team to ensure that the use of established terms did not prevent further inquiry and scrutiny of the data, or that alternative or contradictory meanings could be attributed to it.^37^ All final categories had ‘proven theoretical relevance’ as they were ‘repeatedly present or notably absent when comparing incident after incident’ (p. 177).^37^

Two researchers analysed and coded all data independently to investigate similarities and differences in interpretation, and reduce bias.^35^, ^39^ Data analysis software was not used; data were organized and labelled by hand. Data were coded on their original transcripts for as long as possible to retain context and prevent unnecessary ‘fragmenting’.^35^ To ensure dependability and confirmability a well‐documented and thorough analysis of the whole dataset was conducted, with particular attention paid to disconfirming cases to test and refine devised categories and themes by illuminating limitations and boundaries.^29^ A written summary of the themes was sent to all participants to invite their feedback and comments; no participants responded to this invitation.

## Results

Four core themes described factors that increased or decreased HRQoL in NMO (displayed in Supplementary Figure 2).

Physical symptoms challenged participants' perceptions of normality and prevented the completion of routine activities, which in turn decreased HRQoL. Stability and improvement, through physical recovery, or improvement management of symptoms, increased HRQoL. A method of limiting the impact of physical symptoms on normality was to utilize support in a way that promoted independence. Where participants experienced a loss of prior independence, and experienced increased dependence, their HRQoL decreased, however finding ways to use support to maximize their remaining independence could increase HRQoL.

Living with NMO could decrease individuals' expectations for life (and subsequently HRQoL) where they felt it prevented them from meeting prior goals, expectations and achievements, especially those achieved by their peers. Where individuals felt their ability to fulfil their meaningful roles and meaningful activities was decreased (in turn decreasing their sense of purpose, self‐worth and ultimately HRQoL) this could decrease their future expectations for life. Where participants' sense of living a meaningful and purposeful life with NMO increased, (often due to less influential NMO symptoms and increased support), their expectations for life increased and their HRQoL improved.

These themes are discussed in greater detail below.

### Impact of physical symptoms on normality and HRQoL

NMO symptoms limited HRQoL by preventing people from achieving what they perceived as normality. This was often a direct comparison between what they were previously able to do and what they were now able to do, after the onset of NMO.

Physically I cannot do what I used to be able to do and that impacts on my independence and my ability to do what it is I enjoy doing. Participant 15, female

Specific physical symptoms such as vision loss, pain, continence and limited mobility/dexterity had a negative impact on activities of daily living and lowered HRQoL. They also had further reaching effects through isolation, dependency and lack of social contact.

A good quality of life would be to be pain free. If I was in control of the pain that would make the most difference but that's something that you can't control, can't switch it on and off. Participant 4, female

My wife's out a lot so most times I'm in on my own. I am limited, don't like to admit it, but I'm useless with the stick. Participant 3, male

The severity of symptoms and resultant support needs could prevent any opportunity of living a normal and independent life.

Catheterizing is the main thing, it's the big obstacle for me. So my family want to go away so I've got to go with them because I'd be no good overnight trying to catheterize myself, so I've got to take the mirror and the lamp and we'll need a suitcase like that for all the stuff I need to catheterize. Participant 7, female

I was doing [college course] and cause of when I was in hospital I missed all the work and I couldn't concentrate cause you just have to think and write all the time. With the NMO I wasn't able to function cause I was waking early in the morning and I was always tired, so I just couldn't function with anything they were saying. Participant 2, female

Conversely, an improved HRQoL was perceived as improved health or curing NMO, to enable participants to return to normal activities and things they had previously enjoyed.

[Improved QoL for me] is the consultant to come up with whatever and get my eyesight back. I want to get my life back, I want to go fishing, I want to go to the beach, I want to find friends to hang out with. Participant 14, male

Many participants described HRQoL as having ‘stability’ in both their disease progression and life more generally, as this allowed them the opportunity to adapt and either regain or maintain positive aspects of their life.

I think what HRQoL means to me now is emotional stability with my husband and my marriage, stability in where we're living and having as productive a life as I possibly can in my shrinking world, that is all about quality. Participant 15, female

A major frustration for participants was the loss of control that they experienced due to NMO symptoms.

Poor quality of life is hospitals basically, being away from home, having things that are out of my control, something that will stop me doing what I want to do, something that will stop me getting up. Stop me getting on with my day to day life. Participant 6, female

Conversely, HRQoL was improved where participants felt empowered and able to control situations to enable normal daily life.

### Utilizing support to achieve independence

This theme encompassed the dilemma of maintaining independence, while needing support from others to accomplish desired goals. The level of independence aimed for was very individual, and some goals were more easily achievable than others.

I look at some people going round in wheelchairs with the disease and things I would probably say I've probably still got 10/10 quality of life cos I'm still going out on my own. Participant 12, female

Participants utilized a variety of support including physical (carers), financial (state benefits), health care (adaptations and treatments) and emotional and social support (family and friends) to maximize independence.

I'd say I have quite a good quality of life compared to some people, because I've been lucky in the fact that I've got a really supportive family, really good social support network, so NMO has not stopped me doing anything. I've been supported in going to college and university and working and still go on holiday, I still do a lot of things that I would probably have done before. Participant 6, female

I had a go with a guide dog and decided that was what I wanted. I got a guide dog and that was me on the open road you know. On my own. Didn't have to get towed around by people, because that's still frustrating. Participant 3, male

Decreased independence was strongly linked to a lowered HRQoL, including the loss of financial autonomy and the loss of physical abilities and independence, which could be difficult to adapt to psychologically.

One of the reasons I did a degree was to be financially independent and not to have to rely on anybody so that I'd bring in my own money. That's been another disappointment in itself. Participant 13, female

Participants with severe disability experienced dependency on others for personal care and constant supervision, which decreased their HRQoL.

My mum had a tough time with it when I got out of hospital I think it got worse for them because they realised how much they need to care for me, it's like being a baby again. I had to learn how to walk again, and constantly having to go to the toilet and having accidents. Participant 2, female

The thing I found the hardest is the fact that I will always need somebody there to do things for me, never going to have a day by myself where I can just, get in the car, drive to the shops‐ like normal day to day things really. Participant 6, female

One participant represented the overall sample by showing how perceptions of HRQoL improved as they started to appreciate the things they could still do (even with support), instead of focusing only on skills and activities that were no longer possible.

I feel stripped of my independence and don't feel like I've got much QoL, I've got to rely on people all the time for this, that and the other. But then again I get little sparks that make me think, well hold on, I can still do things, I still talk to people, I still do my gym thing, I still do most things really, just with help now. Participant 13, female

Once participants found ways to use support to maximize their independence, they reported increased HRQoL.

### Expectations for life

Participants described how their plans, goals and expectations had changed since the onset of NMO. They had specific ideas of what constituted their societal expectations, often fitting with gender stereotypes such as marriage and children, through to smaller events such as attending parties and holidays.

I've had to give up work and studying, I gave that up very quickly. I had to give up my career aspirations, that was really a decision that was taken away from me, I know that just maintaining a busy job was going to be enough of a challenge. Participant 15, female

They experienced sadness and frustration when specific NMO symptoms challenged these roles or changed the family dynamic in a negative way.

It's been difficult the past years, everyone's moved on and got their jobs and their circle of friends within jobs and I'm left behind in a way. I'm left where I was and everybody else has moved on, living their lives, so that's really difficult. Participant 8, female

The need to achieve life goals in comparison to peers was reported most commonly by younger women who had experienced NMO relapses as adolescents.

I suppose at 21 I had expectations and an image of what I wanted my life to be. And I just feel like I've missed out on all my twenties and been left behind. Basic things like going abroad, going on holiday. Just even going to parties and just normal things. It's like my life stopped. Participant 8, female

Older participants with NMO had often married or had children before their first major NMO relapse and so were more concerned about maintaining these roles than achieving key goals (e.g. marriage, employment).

### Achieving meaningful roles in life and purpose

Participants described how NMO challenged their role in life and purpose. They felt that NMO decreased the activities that were meaningful to them such as employment and hobbies, preventing normal routines and decreasing their sense of worth and usefulness.

Quality of life? I suppose it's doing like purposeful things and sometimes it feels that is lacking. Sometimes I think what am I getting up for, you know, just to be medicated? Participant 13, female

These feelings were echoed in their social and personal relationships, where NMO prevented individuals fulfilling what they perceived as meaningful roles.

Sometimes I get really upset, I don't like seeing my husband taking the washing out of the machine and things like that, I feel “oh don't do that, that's my job.” Participant 12, female

My fatigue ruins the [marital] relationship, so that role has changed as well with regard to what if my husband and daughter become more like carers a lot of the time, as opposed to me doing the mothering role and me being a fully participating wife? Participant 4, female

For people who were the sole earner in the family, financial concerns were especially pertinent.

Well it's affected me a lot really, what I used to do with sales and exhibitions in my business, and I just can't do it, it puts a lot more pressure on my son. Participant 10, male

I lost my job, I was doing it for 16 years, the mortgage is a big worry on my head, and I know at some time the job center will want to try and stop my incapacity [state] benefit. Participant 1, female

For many people meaningful activities involved socializing with family and friends, while a decrease or loss of friendships and social contact resulted in isolation, lack of self‐fulfilment and purpose. When participants contrasted their life before NMO and their life now, it was often a negative comparison, highlighting lessening independence and a feeling of being left out or left behind in important relationships.

I'd like to be more involved, but my wife likes to stride out and do things (metaphorically as well as physically) and sometimes I feel a bit left behind. Which is frustrating and annoying because for most of my life I wasn't left behind. Participant 5, male

A good quality of life is when you can love someone and you don't have to be embarrassed in situations because of the NMO, cos I've always been the type of person that I wanted to be with someone. I wanted to love someone and I wanted someone to love me and with this right here it's just like it kind of like blew that clean out of the water because I really have to explain myself to everyone I meet. Participant 14, male

These comparisons to past and current sense of self could result in negative perceptions of oneself currently, which lowered HRQoL. Once people felt that their NMO had become more manageable to live with (i.e. stability of condition or increased acceptance) then they reported gaining their sense of purpose and completing activities and social roles more fully again.

Once I know what I've got, what I've got to work with, then I can work with it but the fact that it is so changeable I can't adapt quick enough, but when I know what I've got then I can adapt and then I will be independent again and I will get round things again. Participant 13, female

## Discussion

This study provided the first qualitative investigation of HRQoL in NMO. It outlines a multidimensional conceptual understanding of this topic (as outlined by people with NMO themselves), comprising physical, psychological, social, cultural and environmental facets of well‐being, all with concomitant support needs requiring a complex assortment of formal and informal services.

The findings suggest that maintaining a subjectively high HRQoL requires individuals to perceive their life as meaningful and as close to ‘normal’ as possible. This is achieved by maintaining meaningful roles and activities, maximizing independence, achieving goals in comparison to peers and minimizing the impact of NMO symptoms on daily life.

This patient‐reported emphasis on meaningful roles and activities provides a useful contrast to prior research focusing on the relationship between physical symptoms and HRQoL in NMO,^24^, ^25^, ^26^ as it suggests that a ‘normal’ life in comparison to peers is not solely dependent on physical status, but on achieving desired goals. As such, at a conceptual level HRQoL in NMO hinges around broader concepts of life attainments and activities. In this respect there are conceptual links with Atwal *et al*.^13^ who found that QoL in post‐polio syndrome (a disabling muscular condition developed after an acute attack of polio), was related to ‘happiness’ (defined as enjoyment, satisfaction and excellence). NMO influenced QoL where it prevented enjoyable activities, and limited satisfaction where it lowered competency in valuable activities (excellence) potentially lowering individuals’ feelings of satisfaction.

Participants’ experiences involved subjective perceptions of themselves and their relationships with significant others in their life. A good HRQoL was achieved when NMO had minimum impact on achieving ‘normality’. How people perceived normality was highly varied, and depended on their past experiences and peers, in addition to wider cultural and societal factors. Conceptually this highlights the importance of perceived social comparisons, particularly with age‐matched peers, and demonstrates that the activities and achievements that comprise these attainments are not fixed, but vary between individuals and within individuals at different times of their life.

NMO symptoms had an impact on HRQoL, through the restrictions they imposed on normal daily activities that were important to participants’ identity and well‐being. However, they were not the sole indicators of HRQoL and many people expressed a ‘disability paradox’^40^ where people with severe disability (as measured objectively) reported a high HRQoL, suggesting that it is inappropriate to assume a negative HRQoL on the basis of an individual's neurological impairment.

The importance of expectations has been highlighted previously in the measurement of QoL, where it has been argued that reference values of health and its meaning may change over time, influencing expectations for prognosis and treatment.^41^ Differing expectations, level of adaptation and previous experiences of treatment may also influence perceived HRQoL. The ‘self‐discrepancy’ theory proposed by Higgins^42^ explained that individuals experience distress and discomfort when there is a discrepancy between how they perceive their ‘actual’ self and their perception of their ‘ideal’ self, incorporating their goals, ambitions and expectations for life. In relation to quality of life, this suggests that a low quality of life will be experienced where individuals perceive a large gap between their current situation and sense of self and their idealized view of their desired self.^43^ This theory is in evidence in our study findings by highlighting the incongruence between individuals with NMO's hopes and expectations, and their current experience. Crucially, using this theory in future conceptual frameworks will allow the measurement of individuals’ perceptions of their ability to reach expected goals. Beliefs that expected goals were no longer obtainable after the onset of NMO was highly influential in QoL and conversely regaining belief in their achievability was a key indicator of increased acceptance and adjustment to living with NMO, concurrently with increased QoL. These issues must be incorporated conceptually and methodologically in the development of a self‐reported outcome questionnaire for patients with NMO.

### Limitations of the Study

Although the sample size was small (*n* = 15), it was from a rare population and data saturation was achieved.^28^ The rarity of this condition may limit the transferability to other chronic conditions, however the identified themes are relevant to other conditions which cause physical disability.

The sample was purposively selected to be of different ages, gender, ethnicity and level of disability. Our sample did not include younger men (the age range for men was 52–74) due to an older average age of onset and higher prevalence in women. Therefore, this study cannot be claimed to represent the factors of relevance to their HRQoL, especially given potential gender differences in HRQoL.^44^ The higher proportion of women in this study is potentially attributable to the gender ratio in NMO (females : males; 5 : 1).^2^ Although in some countries NMO has a higher incidence in African American and Asian populations,^45^ the clinical population in which this sample was recruited from has been previously found to be 88% Caucasian.^2^ The majority of participants who opted in to this study were White British, mirroring the demographic of the local clinical population and limiting the conclusions that can be drawn about HRQoL in people with NMO of other ethnic backgrounds.

For this initial study we focused on the lived experience of adults (aged 18 years or over). As there are established differences in the lived experience of children and adults with neurological conditions such as multiple sclerosis,^46^ and potentially differing aspects of QoL, this study cannot provide a conceptual framework for QoL of children living with NMO, however a contemporaneous study by our team investigating this topic is underway.

Health‐care services can play a key role in limiting the negative impact of symptoms and maximizing independence, therefore it is possible that HRQoL is experienced differently in those who do not receive specialist medical services. This could not be investigated through the current study as all participants were recruited through their specialist medical care centre. Identifying potentially discrepant cases (i.e. people who have disengaged from medical services) may further contribute to the understanding of quality of life in NMO.

The necessary decision to exclude participants experiencing relapse, potentially skewed the sample to people whose NMO was stable, thus potentially impacting less on their daily activities and HRQoL.

### Implications for support provision

Management of physical symptoms or compensatory approaches are intrinsic to a good HRQoL. Awareness of individuals at risk of HRQoL deterioration may assist professionals to target interventions most effectively.^47^ These require education of multidisciplinary teams at both a local and national level, involving both specialist NMO services and more generic rehabilitation services.

To gain the optimum balance between support and independence, it may be necessary to include family members or paid carers in the care planning process, however the experiences of informal carers and family members of people with NMO have only recently been explored in a study by our team.

Perceptions of HRQoL and goal achievement were highly personal and subjective. They require a person‐centred approach utilizing shared decision making to ensure that care plans meet the targets relevant to the individual.^48^ Where goals for physical rehabilitation are focused on aims that are currently unachievable (e.g. total physical recovery), psychological interventions targeting evaluation of thoughts and problem‐solving might be of greater benefit, e.g. reframing normality in relation to pain or fatigue.^49^

## Conclusion

This study provides the first qualitative exploration of the factors relevant to HRQoL in people with NMO. We found that, as in other chronic conditions, the impact of physical symptoms, maintenance of meaningful roles and loss of independence were major factors determining HRQoL. However, we also identified additional considerations regarding HRQoL in our NMO study population including the importance of retaining a sense of normality and that positive social comparisons appeared to be equally important and may not be totally dependent on disability severity. Our findings suggested that health‐care professionals from a variety of disciplines are required to provide holistic support to achieve patient chosen goals and maximize HRQoL. These findings will be important considerations in the underpinning HRQoL conceptual framework for a patient‐reported outcome measure in NMO.

## Declaration of conflicting interests

The authors declare that there is no conflict of interest.

## Funding

This work was supported by the Specialist commissioning group of the National Health Service who fund the UK Specialist NMO service.

## Supporting information


**Figure S1** An example of the theme ‘meaningful roles in life and purpose’.
**Figure S2** Thematic diagram of the interconnected bidirectional themes influencing HRQoL in NMO.
