# Supplementary material for: Development of a patient‐centred conceptual framework of health‐related quality of life in neuromyelitis optica: a qualitative study
Source: Health Expect. 2015 Nov 24;20(1):47–58. doi: 10.1111/hex.12432 (PMC5217881; doi:10.1111/hex.12432)
Supplement: Supplementary file 1 — Figure S1 An example of the theme ‘meaningful roles in life and purpose’. Figure S2 Thematic diagram of the interconnected bidirectional themes influencing HRQoL in NMO. [file HEX-20-47-s001.docx]

Figure legends

Figure S1. An example of the theme ‘meaningful roles in life and purpose’

Figure S2. Thematic diagram of the interconnected bidirectional themes influencing HRQoL in NMO

Figure S1. An example of the theme ‘meaningful roles in life and purpose’

Adapting roles

Enjoyment

Achieving purpose and meaning with NMO

Impact of NMO on meaningful activities

Impact of NMO on meaningful relationships

Perceptions of self and self-worth

**Categories**

**Overarching theme**

Usefulness

Identity

Expectations of roles

Negative comparisons with past self

Sense of purpose

Achievement

Values

Inclusion

Busyness

‘Meaningful roles in life and purpose’

**Codes**

Self-worth

Figure S2. Thematic diagram of the interconnected bidirectional themes influencing HRQoL in NMO

Quality of life in NMO

**Expectations for life**

- Prior expectations
- The emotional impact of unfulfilled expectations
- Achieving goals in comparisons to peers
- Disrupted plans, goals and expectations

**Utilising support to achieve independence**

- Loss of prior independence
- The role of support
- Living with dependence
- Maximising independence

NB. All themes interconnect in the lived experience of HRQoL in NMO. All themes influence HRQoL bidirectionally, represented by double-headed arrows.

**Impact of physical symptoms on normality and HRQoL**

- Perceptions of normal life
- Impact of NMO symptoms on activities
- Stability and improvement
- Controlling and managing symptoms

**Achieving meaningful roles in life and purpose**

- Perceptions of self and self-worth
- Impact of NMO on meaningful activity
- Impact of NMO on meaningful relationships
- Achieving meaning and purpose with NMO
